# Mesenchymal stem cells as carriers and amplifiers in CRAd delivery to tumors

**DOI:** 10.1186/1476-4598-10-134

**Published:** 2011-11-03

**Authors:** Xi Xia, Teng Ji, Pingbo Chen, Xiao Li, Yong Fang, Qinglei Gao, Shujie Liao, Lanying You, Hongbin Xu, Quanfu Ma, Peng Wu, Wencheng Hu, Mingfu Wu, Li Cao, Kezhen Li, Yanjie Weng, Zhiqiang Han, Junchen Wei, Ronghua Liu, Shixuan Wang, Gang Xu, Daowen Wang, Jianfeng Zhou, Ding Ma

**Affiliations:** 1 Cancer Biology Research Center, Tongji Hospital, Tongji Medical College, Huazhong University of Science and Technology, Wuhan, Hubei 430030, P. R. China; 2 Department of Gynecology & Obstetrics, Affiliated Shenzhen Nanshan Hospital, Guangdong Medical College, Shenzhen, Guangdong 518052, P. R. China; 3 Department of Pediatrics, Maternal and Child Health Hospital of Shenzhen, Southern Medical University, Shenzhen, Guangdong 518038, P. R. China; 4 Department of Gynecology, Cancer Hospital of Fujian, Fujian Medical College, Fu Zhou, Fujian 350014, P. R. China; 5 Department of Gynecology & Obstetrics, People's Hospital of Shenzhen, Shenzhen, Guangdong, 518020, China

**Keywords:** Mesenchymal Stem Cell, Conditionally Replicative Adenovirus, Cell Carrier, Signal Transducer and Activator of Transcription 3 (Stat3), Breast cancer

## Abstract

**Background:**

Mesenchymal stem cells (MSCs) have been considered to be the attractive vehicles for delivering therapeutic agents toward various tumor diseases. This study was to explore the distribution pattern, kinetic delivery of adenovirus, and therapeutic efficacy of the MSC loading of E1A mutant conditionally replicative adenovirus Adv-Stat3(-) which selectively replicated and expressed high levels of anti-sense Stat3 complementary DNA in breast cancer and melanoma cells.

**Methods:**

We assessed the release ability of conditionally replicative adenovirus (CRAd) from MSC using crystal violet staining, TCID_50 _assay, and quantitative PCR. In vitro killing competence of MSCs carrying Adv-Stat3(-) toward breast cancer and melanoma was performed using co-culture system of transwell plates. We examined tumor tropism of MSC by Prussian blue staining and immunofluorescence. In vivo killing competence of MSCs carrying Adv-Stat3(-) toward breast tumor was analyzed by comparison of tumor volumes and survival periods.

**Results:**

Adv-Stat3(-) amplified in MSCs and were released 4 days after infection. MSCs carrying Adv-Stat3(-) caused viral amplification, depletion of Stat3 and its downstream proteins, and led to significant apoptosis in breast cancer and melanoma cell lines. In vivo experiments confirmed the preferential localization of MSCs in the tumor periphery 24 hours after tail vein injection, and this localization was mainly detected in the tumor parenchyma after 72 hours. Intravenous injection of MSCs carrying Adv-Stat3(-) suppressed the Stat3 pathway, down-regulated Ki67 expression, and recruited CD11b-positive cells in the local tumor, inhibiting tumor growth and increasing the survival of tumor-bearing mice.

**Conclusions:**

These results indicate that MSCs migrate to the tumor site in a time-dependent manner and could be an effective platform for the targeted delivery of CRAd and the amplification of tumor killing effects.

## Background

Malignant tumor remains one of the leading causes of mortality throughout the world, and new treatments are urgently needed [1-3]. In addition to surgery, radiotherapy, and chemotherapy, oncolytic viruses have emerged in recent years as a promising novel class of therapeutics for the treatment of human cancers [4-6]. Among these viruses, conditionally replicative adenoviral agents (CRAd) have been designed to replicate in tumor cells, whereby the virus can self-amplify and spread in the tumor from an initial infection of only a few cells [7-9]. In our previous studies, we constructed many mutants of CRAd and validated their selective antitumor activities [10-12]. However, the effective use of CRAd in clinical applications has been limited by sequestration in the liver and reticuloendothelial system [13-15], inefficient virus delivery to the tumor site [6,16,17], and rapid elimination as a result of immune clearance [18-20]. Thus, several approaches have been exploited to overcome the limitations, including genetic alteration of the virus [21,22], nanotechnology modifications [23], and the application of cell carriers, such as cytotoxic T-lymphocytes [20], neural stem cells [24,25], and mesenchymal stem cells (MSCs) [26-28]. Among these cell carrier systems, MSCs are considered to be the most attractive candidate for viral delivery to the tumor site due to ethical and technical convenience, poor immunogenicity, and genetic stabilization [29]. MSCs have been shown to migrate toward malignant tumors [26,28-30] and track microscopic metastasis [27], while the molecular mechanism underlying the tumor-directed migration of MSCs has not been completely elucidated [31]. MSCs engineered with CRAd have been indicated to deliver viruses into lung metastases of breast carcinoma [28], ovarian cancer [4], and intracranial glioma [26,32]. However, information regarding the kinetic process underlying MSC trafficking to tumors, its in vivo distribution pattern, and the release of adenovirus carrying anti-sense complementary DNA from MSCs is still lacking.

In the present study, we investigated the ability of MSCs to deliver an E1A mutant CRAd targeting Stat3 to orthotopic breast cancer. The E1A mutant was engineered with a 27-bp deletion in the E1A CR2 region necessary for retinoblastoma (Rb) protein binding to attenuate viral replication and cytolysis in normal quiescent tissues but not in tumor cells [11,12]. We demonstrate that Adv-Stat3(-) can be loaded by MSCs, exponentially amplified up to a certain limitation, and eventually released. In vivo experiments show that intravenous injections of MSCs loaded with Adv-Stat3(-) can preferentially home to the tumor site, and the number of homing cells increases gradually with time. Moreover the Adv-Stat3(-) engineered MSCs carries anti-sense target complementary DNA to the tumor site and suppresses Stat3 expression and its downstream molecules, thus inhibiting tumor growth and increasing the survival of tumor-bearing mice. Taken together, we show that MSCs represent an amplification site for E1A mutant CRAd, allowing previous viral stocks to be protected until it can be delivered and released into the tumor in a time-dependent manner.

## Methods

### Cells Isolation and Culture

Human MSCs were isolated from the bone marrow of normal individuals undergoing bone marrow harvest for allogeneic bone marrow transplantation in our department (Hematological Center, Tongji Hospital, Huazhong University of Science and Technology) after obtaining informed consent according to institutional guidelines under the approved protocol. Briefly, mononuclear cells were separated by centrifugation over a Ficoll-Hypaque gradient (Sigma Chemical Co., St. Louis, MO) and suspended in α-MEM medium containing 20% fetal bovine serum (Life Technologies, Inc., Rockville, MD), L-glutamine, and penicillin-streptomycin. After 3 days, the non-adherent cells were removed by washing with PBS, and the monolayer of adherent cells were cultured until they reached confluence. Cells were trypsinized using tryple™ express (Invitrogen Co., Carlsbad, CA) and used for experiments at passages three to eight. For identification, the cells were trypsinized, washed with PBS, and then reacted with FITC-labeled anti-CD34, PE-labeled anti-CD44, FITC-labeled anti-CD45, FITC-labeled anti-CD19, PE-labeled anti-CD90, and PE-labeled anti-CD105 antibodies (Biolegend, San Diego, CA, USA). After washing with PBS, the expression level of these molecules was determined by flow cytometry. The isotope antibodies were used as negative controls.

Human umbilical vein endothelial cells (HUVEC) were isolated and cultured as described previously [33]. The human embryonic kidney 293, MDA-MB-231 breast cancer cell line, and MDA-MB-435 melanoma cell line [34] were obtained from the American Type Culture Collection (ATCC) in 2008 and maintained in DMEM and RPMI-1640 medium containing 10% FBS, L-glutamine, and penicillin-streptomycin at 37°C in a humidified 5% CO_2 _atmosphere.

### In Vitro Differentiation of MSCs

For adipogenic differentiation, the MSCs were maintained in medium containing 1 μM dexamethasone, 0.35 mM hydrocortisone, 100 μg/mL 3-isobutyl-1-methylxanthine, 0.1 μg/mL insulin, and 60 μM indomethacin. Two weeks later, the cells were fixed and stained with Oil-red O (Sigma). Osteoblast differentiation was performed by culturing MSCs in medium plus 10 μM dexamethasone, 0.1 mM ascorbic acid, and 10 mM β-glycerophosphate. Three weeks later, the cells were washed, fixed, and stained with Alizarin red. To induce chondrogenic differentiation, MSCs were pelleted and maintained in medium with 2% FBS and 10 ng/mL TGF-β_3_. After 14 days, the pellets were formalin-fixed, frozen, and cryostat sections were stained with Alcian blue (Sigma).

### Recombinant Adenoviruses

The conditionally replicative adenovirus Adv-anti-sense Stat3 (Adv-Stat3(-)) was driven from Adenovirus 5 through the deletion of amino acids 121-129 in E1A CR2 and the replacement of ADP open reading frame in the E3 region by a fragment of reverse Stat3 cDNA (bases 960-190) (12). Replication-defective adenovirus Adv-GFP was described previously [35]. The wild-type adenovirus Adv-wt was obtained from ATCC.

### Recombinant Adenovirus Release from MSCs In Vitro

MSCs plated in 24-well plates were infected with adenovirus. At indicated time points, the cells were fixed and stained with 1% crystal violet in 4% formaldehyde for 20 minutes, followed by washing with tap water to remove excess dye. Plate images were captured with a Kodak DC260 digital camera (Kodak, Rochester, NY).

For the viral replication assay, the infected MSCs were harvested at indicated time points and cell lysates were prepared to isolate the viral DNA and total cell DNA using the QIAamp DNA Blood kit (Qiagen, Valencia, CA). Real-time PCR targeting the viral fiber region was performed to quantitatively evaluate the adenoviral DNA copy number. An internal GAPDH control was used to normalize the results. The primer sequences targeting fiber and GAPDH were as follows: fiber sense, 5' -AAA TGT GGC AGT CAA ATA C-3'; fiber anti-sense, 5'-ATA GGT TAG GCA TAA ATC C-3'; GAPDH sense, 5'-ACG GAT TTG GTC GTA TTG GG -3'; GAPDH anti-sense, 5'-TGA TTT TGG AGG GAT GTC GC -3'.

### TCID_50_

Viral yield was determined by the limiting dilution method, known as the determination of 50% tissue culture infective dose, described previously [12]. The data from three separate infection studies were expressed as plaque-forming units per milliliter.

### In Vitro Cancer Cell Cytotoxicity of Adenovirus Loaded MSCs

MDA-MB-231, MDA-MB-435, and HUVEC cells were trypsinized and plated in the lower chambers of transwell plates; PBS, MSC, Adv-Stat3(-), and MSC/Adv-Stat3(-) were added to the upper chambers of the transwell plates. After 3 days, the cells in the lower chamber were harvested and stained using the annexin-V/PI apoptosis kit (Bender MedSystems, Vienna, Austria) according to the manufacturer's instructions. Apoptotic cells were counted using a FACScan flow cytometer. The data were analyzed using cell fit software.

### In Situ Hybridization

Slides were pre-hybridized for 30 min at 37°C. Hybridization was carried out overnight at 42°C with 1 μg/mL rhodamine-conjugated viral fiber oligonucleotide probe complementary to the fiber coding region (5-GGA ACT GGC CTT AGT TTT GAC AGC ACA GGT GCC ATT ACA G-3) [35]. Negative controls were similarly processed by omitting the probe. The results were observed under a fluorescent microscope.

### Western Blot Analysis

Total proteins were extracted by lysing the cells in buffer containing 50 mM Tris (pH 7.4), 150 mM NaCl, 0.5% NP-40, 50 mM NaF, 1 mM Na_3_VO_4_, 1 mM phenylmethylsulfonyl fluoride, 25 mg/mL leupeptin, and 25 mg/mL aprotinin. The lysates were cleared by centrifugation and the supernatants collected. Equal amounts of protein lysate were used for Western blot analyses using the indicated antibodies. Specific signals were visualized with NBT/BCIP.

### Mouse Tumor Model

Female athymic BALB/c mice (3 to 4 weeks old) were obtained from the Animal Experimental Center (Shanghai, China). The mice were used in accordance with institutional guidelines under the approved protocols. MDA-MB-231 cells (2 × 10^6^) were suspended in 100 μL PBS and injected into the mammary fat pad of nude mice. After 20 days, most mice grew an orthotopic tumor approximately 4 mm in diameter. After the indicated treatments, tumors were measured with calipers in two dimensions every 4 days, and the volume was calculated as length × width^2 ^× 0.52.

### Survival Analysis

Twenty days after MB-MDA-231 tumor cell implantation, the mice were injected with 1 × 10^6 ^MSC/Adv-Stat3(-) (n = 8), an equivalent dose of Adv-Stat3(-) (n = 8) and MSCs (n = 8). Negative control mice (n = 8) received PBS. All mice were followed daily until death. None of the mice had to be sacrificed because of excessive bleeding, open wound infection, moribund status, or cachexia. Survival data were plotted on a Kaplan-Meier curve. The difference in survival was determined by the log rank test.

### Immunohistochemistry

Immunohistochemical analyses of frozen sections were performed as described previously [36].

### Statistics Analyses

All experiments were repeated three times unless otherwise specified. Data were analyzed using independent sample *t *tests and are expressed as mean ± SD. A p-value < .05 was considered significant. Statistical analyses were performed using SPSS software version 13.0 (SPSS Inc., Chicago, IL).

## Results

### Characterization of MSCs and the Transfection Efficiency of Adenovirus

**Figure 1:**
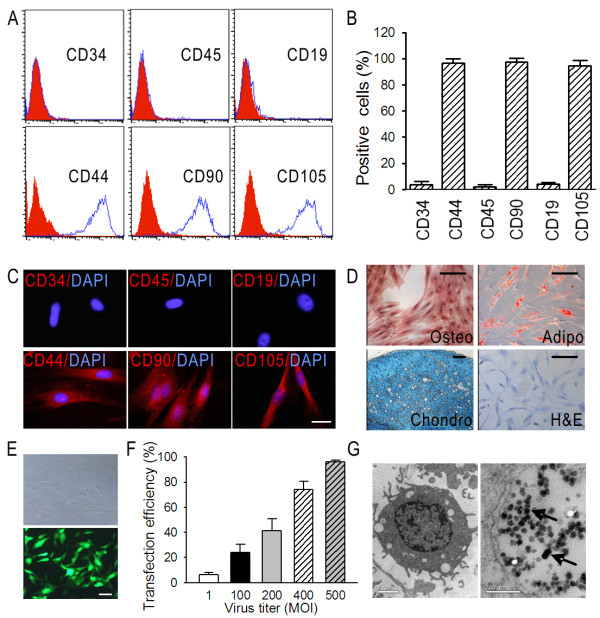
**Identification of expanded human MSCs and adenovirus transfection efficiency**. (A) Representative histogram overlays of FACS analysis showing the MSC antigen profile. The blue peak represents the specific PE-labeled antibodies: CD34, CD45, CD19, CD44, CD90, and CD105. The red peak represents the corresponding isotope antibodies. (B) Positive expression of CD34, CD45, CD19, CD44, CD90, and CD105 in five independent samples was expressed as mean ± SD as evaluated by FACS. (C) Immunostaining of CD34, CD45, CD19, CD44, CD90, and CD105 in one representative sample as observed under a confocal microscope. Negative controls were performed by using the corresponding isotope antibodies. Red, PE-labeled antibodies; blue, DAPI. (D) Representative photographs showing the in vitro differentiation of MSCs into the osteogenic, adipogenic, and chondrogenic lineages. Undifferentiated MSCs were dyed with hematoxylin and eosin. (E) Representative images depicting the transfection of MSCs with 500 MOI Adv-GFP. Forty-eight hours after transfection, MSCs carrying GFP were observed under bright field (upper panel) and fluorescent field (lower panel). (F) Transfection efficiencies at different Adv-GFP titers after 48 hours in three independent MSC samples were expressed as mean ± SD as evaluated by FACS. (G) Electron micrographs showing viral particles in MSCs. MSCs were infected with 500 MOI Adv-Stat3(-). Seventy-two hours later, MSCs were collected and prepared to be observed under an electron microscope. Adenovirus particles in the MSCs were shown using the black arrow. C, scale bar = 5 μm; D, scale bar = 10 μm; E, scale bar = 10 μm; G, left scale bar = 2 μm, right scale bar = 200 nm).

Given the lack of a specific marker in MSCs [31,37], flow cytometry and immunofluorescence were used to validate that the isolated cells were negative for typical hematopoietic antigens CD34, CD45, and CD19 and positive for CD44, CD90, and CD105 (Figure 1A and 1C). The proportion of cells expressing CD34, CD45, CD19, CD44, CD90, and CD105, which were analyzed from 5 independent samples, were 3.8% ± 2.3%, 2.2% ± 1.5%, 4.0% ± 1.3%, 96.6% ± 3.2%, 97.5% ± 2.1%, and 94.1% ± 4.1% (Figure 1B). The MSCs had a differentiation capability for osteogenesis, chondrogenesis, and adipogenesis (Figure 1D).

We tested the transfection efficiency of the MSCs using the replication-defective vector Adv-GFP. The representative transfection image of 500 multiplicity of infection (MOI) is shown in Figure 1E. Flow cytometry revealed the efficiency at 48 hours after transfection was dose-dependent and reached 96.4% ± 1.2% at 500 MOI (Figure 1F). To directly verify the E1A-mutant CRAd loading capability, the MSCs were infected with 500 MOI Adv-Stat3(-). Seventy-two hours after infection, electron microscopy verified the presence of intracellular viral particles in the MSCs (Figure 1G).

### Adv- Stat3(-) Exponentially Amplified in MSCs and Were Eventually Released

**Figure 2:**
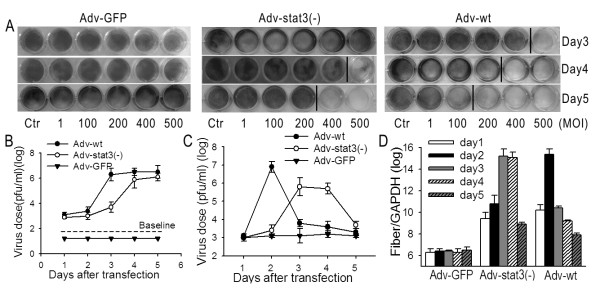
**Adv-Stat3(-) replicates adequately in MSCs and is eventually released**. (A) Cytopathic effects of Adv-GFP, Adv-Stat3(-), and Adv-wt on MSCs. MSCs were infected with Adv-GFP, Adv-Stat3(-), and Adv-wt at different MOI. The cytotoxic activity was evaluated by crystal violet staining at each time point. The control without any viral infection is indicated as Ctr. (B) Viral count in the culture medium of MSCs was expressed as mean ± SD in three independent experiments. MSCs were infected with 500 MOI Adv-GFP, Adv-Stat3(-), and Adv-wt. At each time point, the culture medium was collected and the viral concentration measured using the TCID_50 _method. (C) Intracellular viral concentration was expressed as mean ± SD in three independent experiments. MSCs were infected with 500 MOI Adv-GFP, Adv-Stat3(-), and Adv-wt. At each time point, the intracellular virus was extracted by three cycles of freezing and thawing and measured using the TCID_50 _method. (D) DNA replication of Adv-GFP, Adv-Stat3(-), and Adv-wt in MSCs. MSCs were infected with 500 MOI Adv-GFP, Adv-Stat3(-), and Adv-wt. At each time point, MSCs were harvested and real-time PCR performed to measure the amount of viral DNA. Results are the means of three independent experiments and expressed as the ratio with GAPDH. Negative controls omitted templates.

After confirming MSCs could efficiently load Adv-Stat3(-), we aimed to assess whether and when the adenovirus is released from the cell using Adv-GFP, Adv-wt, and Adv-Stat3(-). We hypothesized the release of adenovirus was a result of MSC lysis. Crystal violet staining showed Adv-Stat3(-) up to 500 MOI was released from MSCs after 4 days, and this capability was stronger on day 5 (Figure 2A). The negative control for Adv-GFP did not display any competence for release, whereas Adv-wt had a much stronger release capability than Adv-Stat3(-). Further analysis using the TCID_50 _method to examine the amount of virus released from MSCs found the amount of virus released into the culture medium markedly increased from day 3 (10^3.7^pfu/mL) to a high level of 10^5.9^pfu/mL on day 4 when MSCs were incubated with 500 MOI Adv-Stat3(-). The amount of virus released remained steady until day 5 (10^6.1^pfu/Ml)(Figure 2B); In the wild-type group, the concentration in the culture medium displayed a parallel, but earlier, increase compared to Adv-Stat3(-), and virus was not detected in the culture medium for the Adv-GFP group. The amount of intracellular adenovirus in MSCs was also measured. The amount of virus in Adv-Stat3(-) group greatly increased from 10^3.0^pfu/mL on day 1 to 10^5.8^pfu/mL on day 3 and remained invariable between days 3 and 4, eventually decreasing to 10^3.7^pfu/mL on day 5 (Figure 2C). The amount in the Adv-wt group exhibited an earlier, but parallel change as Adv-Stat3(-). The amount of virus in the Adv-GFP group was unchanged during this period.

In addition, quantitative PCR to detect targeting fiber, a late structural viral gene whose expression is dependent on active viral replication, was performed. Adv-Stat3(-) replicated in MSCs from day 1, and this replication ended at a low level on day 5 (Figure 2D). In accordance with the TCID_50 _experiments, Adv-wt replicated faster than Adv-Stat3(-), and Adv-GFP did not replicate.

### Adv-Stat3(-)-loaded MSCs Selectively Exert an In Vitro Oncolytic Effect on Breast Cancer and Melanoma Cells

**Figure 3:**
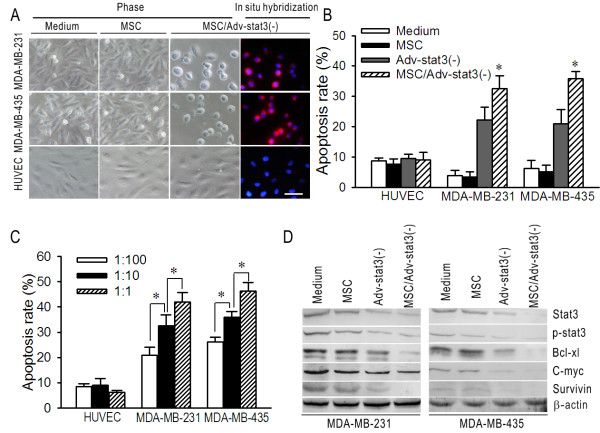
**MSC/Adv-Stat3(-) selectively exerts in vitro oncolytic effects on breast cancer and melanoma cell lines and suppresses the Stat3 pathway**. (A) Cytopathic effects and virus replication induced by MSC and MSC/Adv-Stat3(-) in MDA-MB-231, MDA-MB-435, and HUVEC cells. 1 × 10^5 ^MDA-MB-231, MDA-MB-435, and HUVEC cells were plated in the lower chambers of transwell plates. 1 × 10^4 ^MSCs pre-infected with 500 MOI Adv-Stat3(-) for 48 hours or MSCs alone were added in the upper chambers. Phase contrast images of the cells in the lower chamber were taken after 72 hours and in situ hybridizations targeting viral fiber were performed. Scale bar = 10 μm. Cell nucleuses were counterstained with DAPI. (B) Apoptosis rates caused by MSC, Adv-Stat3(-), and MSC/Adv-Stat3(-) in MDA-MB-231, MDA-MB-435, and HUVEC cells. 1 × 10^5 ^MDA-MB-231, MDA-MB-435, and HUVEC cells were plated in the lower chambers of transwell plates. 1 × 10^4 ^MSCs pre-infected with 500 MOI Adv-Stat3(-) for 48 hours, equivalent Adv-Stat3(-), or MSCs were added in the upper chambers. The cells in the lower chambers were stained with Annexin-V/PI after 72 hours and analyzed by FACS. (C) Apoptosis in the lower chambers in response to different ratios of MSC/Adv-Stat3(-). (D) Representative Western blot demonstrating the changes in Stat3, p-Stat3, Bcl-xl, c-myc, and Survivin caused by MSC/Adv-Stat3(-), Adv-Stat3(-), and MSC. Medium was used as a negative control. *P < .05. Results were expressed as mean ± SD in three independent experiments.

To test the ability of MSCs to deliver CRAd to breast cancer and melanoma cells, a co-culture system using transwell plates was used. After 72 hours, MSC/Adv-Stat3(-) induced notable cytopathic effects (CPE) in MDA-MB-231 and MDA-MB-435 cells, but not in HUVEC cells. The positive CPE in MDA-MB-231 and MDA-MB-435 were concomitant with viral replication evaluated by in situ hybridization. However, MSC *per se *treatment exhibited no detectable CPE in MDA-MB-231, MDA-MB-435, and HUVEC cells (Figure 3A). Flow cytometric assays using the same co-culture system compared the apoptosis caused by MSC, Adv-Stat3(-), and MSC/Adv-Stat3(-) treatment. After MSC/Adv Stat3(-) treatment, 32.5% ± 4.3% and 35.9% ± 2.3% of MDA-MB-231 and MDA-MB-435 cells, respectively, were apoptotic (Figure 3B). Adv-Stat3(-) at the same dose caused apoptosis in 22.2% ± 4.3% and 20.9% ± 4.8% of MDA-MB-231 and MDA-MB-435 cells, respectively, which is significantly lower than the rates seen with MSC/Adv-Stat3(-) (*P *< .05). MSC *per se *treatment did not exert an obvious apoptosis effect on MDA-MB-231 or MDA-MB-435 cells. Significant apoptosis was not induced in normal HUVEC cells by any of the treatments. The co-culture experiments evaluated by flow cytometry were repeated with different ratios of MSC/Adv-Stat3(-) in tumor and HUVEC cells. Apoptosis of the cancer cells significantly increased with an increasing ratio from 1:100 to 1:1 (*P *< .05) (Figure 3C); whereas HUVEC cells continued to have only low levels of apoptosis at any ratio tested.

Immunoblot analysis of the whole cell lysate from the lower chambers showed that MSC/Adv-Stat3(-) reduced the expression of Stat3, p-Stat3, and its downstream target proteins, including Bcl-xl, c-myc, and Survivin, to a greater extent than Adv- Stat3(-) in MDA-MB-231 and MDA-MB-435 cells. However, no obvious change was found for MSC treatment compared to negative control (Figure 3D). In HUVEC cells, none of the treatments produced significant depletion of Stat3 expression (Additional file 1, Figure S1).

### Adv-Stat3(-)-loaded MSCs Preferentially Home to Tumors in a Time-Dependent Manner

To confirm that Adv-Stat3(-)-loaded MSCs preferentially engraft in tumors, we examined the homing capability of MSCs without loading with virus. Twenty days after the establishment of a breast orthotopic xenograft, the mice were intravenously injected with 1 × 10^6 ^SPIO-labeled MSCs. After 24 or 72 hours, the mice were sacrificed and assessed for the presence of MSCs in various tissues using Prussian blue staining (Additional file 2, Figure S2A). MSCs were mainly localized to the tumor periphery at 24 hours. At 72 hours, MSCs were mostly found in the tumor parenchyma (Additional file 2, Figure S2B). However, no normal organs (i.e. kidney, muscle, intestines, contralateral normal breast, or heart) showed signs of MSC engraftment, except for the rare existence in the lung, liver, and spleen (Additional file 2, Figure S2C).

**Figure 4:**
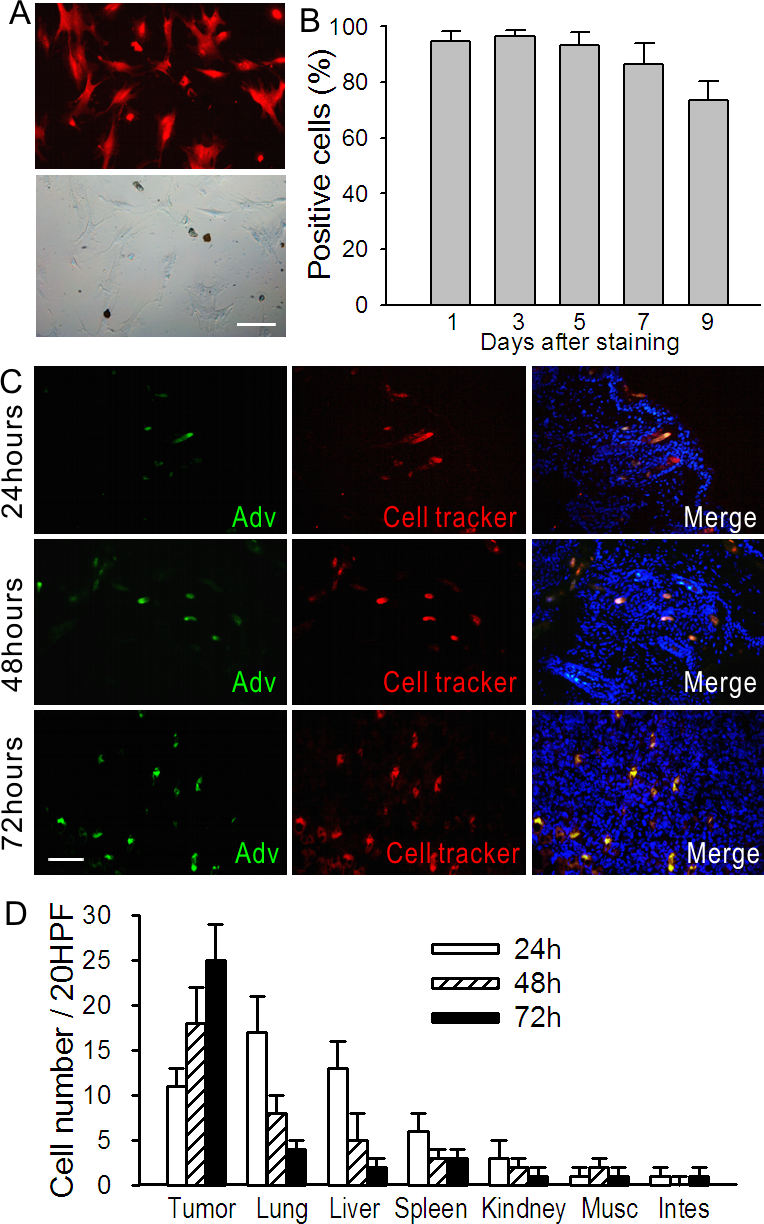
**Kinetic distribution of Adv-Stat3(-)-loaded MSCs in a breast orthotopic tumor model**. (A) MSCs labeled with 5 μM Cell Tracker Red. The cells were observed under a fluorescent microscope after 24 hours (upper panel). The corresponding bright field is shown in the lower panel. Scale bar = 15 μm. (B) Labeling efficiencies of MSCs with Cell Tracker Red at the indicated time points as evaluated by FACS were expressed as mean ± SD in three independent experiments. (C) Representative images showing the distribution of Adv-Stat3(-)-loaded MSCs in an orthotopic tumor. 1 × 10^6 ^Cell Tracker Red labeled MSCs were infected with 500 MOI Ade-Stat3(-). Six hours later, these cells were intravenously injected into tumor-bearing mice. The mice were sacrificed and frozen sections of tumor prepared and incubated with FITC-labeled adenovirus hexon antibody after 24 (upper panels), 48 (middle panels), and 72 (lower panels) hours. The sections were counterstained with DAPI and observed under a fluorescent microscope. Scale bar = 20 μm. (D) Cell number of MSCs in normal tissue from the mice in panel c at the indicated time points. After incubating frozen sections with FITC-labeled adenovirus hexon antibody, the sections were observed under a fluorescent microscope. The number of MSCs were counted under ×20 HPF in five random fields and expressed as mean ± SD (n = 3).

Cell Tracker Red was introduced to track MSCs in vivo and ascertain the tumor tropism of Adv-Stat3(-)-loaded MSCs. One day after in vitro labeling with Cell Tracker Red, the MSCs were found to display a strong red fluorescence (Figure 4A). The labeling efficiencies at different time points were measured by flow cytometry and there were over 90% Cell Tracker Red positive MSCs in the first 5 days. After 9 days, we still found high efficiency of more than 70% (Figure 4B), which supports the sensitivity of in vivo tracking. Next, we injected 1 × 10^6 ^Adv-Stat3(-)-loaded MSCs labeled with Cell Tracker Red into the tumor-bearing mice. The mice at each time point (n = 3 each; 24, 48, and 72 hours after treatment) were sacrificed and examined for the presence of MSCs using a fluorescence microscope. MSCs were identified by red fluorescence, whereas intracellular adenoviruses were examined using FITC-labeled adenovirus hexon antibody, which fluoresced green. MSCs carrying Adv-Stat3(-) appeared as orange in the overlay (Figure 4C). A significant increase in the number of Adv-Stat3(-)-loaded MSCs occurred with time (Figure 4D). At 24 hours, we observed considerable localization of Adv- Stat3(-)-loaded MSCs in the lungs, liver, and spleen, but it sharply decreased to quite a low level by 72 hours. Few Adv-Stat3(-)-loaded MSCs were detected in other tissues, including the kidneys, muscle, and intestines, during this period.

### Adv-Stat3(-)-loaded MSCs Inhibit Breast Tumor Growth in Mice

**Figure 5:**
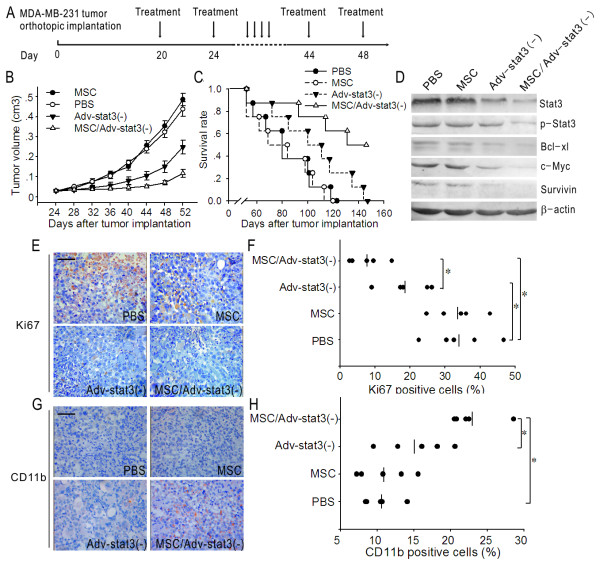
**MSC/Adv-Stat3(-) inhibits tumor growth and improves survival rates in tumor-bearing mice**. (A) Schematic graph showing the design of the in vivo experiment. (B) Tumor growth in mice treated with MSC, Adv-Stat3(-), or MSC/Adv-Stat3(-). Tumor volume in each group was expressed as mean ± SD. (n = 5) (C) Survival curves following the administration of MSC, Adv-Stat3(-), or MSC/Adv-Stat3(-). (D) Representative Western blot analysis of the Stat3 pathway in orthotopic tumors on day 52 after the last measurement of the tumor. (E) Representative immunohistochemistry images showing Ki67-positive cells (brown) in orthotopic tumors on day 52 after the last measurement of tumor. (F) Statistical analysis of Ki67-positive cells counted in five random fields from panel E. (G) Representative immunohistochemistry images showing CD11b-positive cells (brown) in orthotopic tumors on day 52 after the last measurement of tumor. (H) Statistical analysis of CD11b-positive cells counted in five random fields from panel G. *P < .05, E and G, scale bar = 20 μm.

Based on the above results, we hypothesized that the administration of Adv-Stat3(-)-loaded MSCs allows for selective delivery of virus to breast tumor in vivo, improving the therapeutic effect over virus alone. Twenty days after the establishment of breast orthotopic xenografts, we intravenously injected 1 × 10^6 ^Adv-Stat3(-)-loaded MSCs (n = 5 mice; equivalent Adv-Stat3(-) and MSCs) at 4-day intervals for 8 doses. Tumor-bearing mice that received PBS were used as controls (n = 5; Figure 5A). Compared to the control group, tumor growth was inhibited by Adv-Stat3(-) but not MSC treatment. At day 52, the mean tumor volume in the Adv-Stat3(-) group was 248.3 ± 34.2 mm^3^, which was significantly smaller than that of the PBS (437.9 ± 36.3 mm^3^) and MSC (485.4 ± 31.6 mm^3^) groups (*P *< .05). However, when compared to the MSC/Adv-Stat3(-) group, the tumor growth in the Adv-Stat3(-) groups tended to be larger. The mean tumor volume in the Adv-Stat3(-) and MSC/Adv-Stat3(-) groups at day 52 was 248.3 ± 34.2 mm^3 ^and 116.5 ± 20.1 mm^3^, respectively (*P *< 0.05; Figure 5B). After measuring the tumor for the last time, the mice in each group were sacrificed and the tumor masses separated and analyzed by Western blot and immunohistochemistry. In accordance with the in vitro results, MSC/Adv-Stat3(-) obviously reduced Stat3, p-Stat3, and its downstream proteins compared to PBS. Only a slight reduction in Stat3, p-Stat3, and its downstream proteins was observed in the Adv-Stat3(-) group. However, we found no change in the expression of the Stat3 pathway upon MSC treatment (Figure 5D). Immunohistochemical analysis revealed notably fewer Ki-67-positive proliferating cells in MSC/Adv-Stat3(-)-treated mice than mice treated with Adv-Stat3(-) (*P *< .05). Also, a significant reduction in Ki-67-positive cells was observed in Adv-Stat3(-)-treated mice compared to control mice (*P *< .05). However, no significant difference in the number of Ki-67-positive cells was found between the MSC and control groups (Figure 5E and 5F). In contrast, a significantly greater number of CD11b-positive cells which represented granulocytes and macrophages were found in the MSC/Adv-Stat3(-) group than the control group. Although the mice in the Adv-Stat3(-) group had a higher number of CD11b-positive cells than the control group, it was not significant (*P *> .05). Also, no significant difference was found between the MSC and PBS groups (*P *> .05; Figure 5G and 5H).

### Adv-Stat3(-)-loaded MSCs Improve the Survival of Breast Tumor-bearing Mice

Finally, we examined whether Adv-Stat3(-)-loaded MSCs improve the survival of tumor-bearing mice. Mice were intravenously treated with PBS (n = 8), MSC (n = 8), Adv-Stat3(-) (n = 8), or MSC/Adv-Stat3(-) (n = 8) and followed to determine survival rates. The mice treated with MSC/Adv-Stat3(-) survived significantly longer than the other three groups (*P *< .05; Figure 5C). At the end point of the follow-up, four mice treated with MSC/Adv-Stat3(-) were alive, but no mice were alive in the other three groups. In addition, the mice treated with Adv-Stat3(-) but not MSC survived longer than PBS-treated mice (*P *< .05).

## Discussion

The capability of MSCs to incorporate into tumors as a stromal element allows the stem cell to release anti-tumor agents from within the tumor [27,30,38]. Recently, MSCs have been reported to deliver CRAd to various malignant tumors [26,28,32]. However, the kinetic process of CRAd-engineered MSC trafficking to tumors and the release of adenovirus from MSCs is still lacking. To be delivered by a cellular vehicle, CRAd should be capable to efficiently transduce MSCs. Adam [26] and Komarova [4] reported that adenoviral vectors bearing 5/3 or argininine-glycine-aspartic acid (RGD) modifications on their fiber knobs, instead of wild-type 5 knob, are capable of effectively transducing MSCs. These findings are consistent with the result that MSCs had scarce expression of coxsackie and adenovirus receptor (CAR), which immediately mediates the transduction of wild-type adenovirus 5 [39]. However, our findings indicated that ADV with wild-type 5 knob at MOI of more than 500 MOI can be highly transduced in MSCs. We speculated that the contradictive results may be ascribed to the different virus titers used or other unknown molecules other than CAR mediating the transduction. In the present study, we found that E1A-mutant Adv-Stat3(-) at 500 MOI infection exponentially amplified in MSCs up to the utmost quantity in a sufficient time window, which consequently boosted the tumor apoptosis rate compared to direct virus treatment. The results can be explained two ways. First, MSC could function as a manufacturer of E1A-mutant CRAd, allowing the total amount of virus required for therapeutic effects to decrease compared to direct equivalent virus injection. Second, the superior therapeutic response from treatment with Adv-Stat3(-)-loaded MSCs could be due to not introducing too much CRAd initially, which may lead to premature release of adenovirus before it is delivered to the tumor site, and avoid too little CRAd, which may restrict the maximal tumor oncolysis.

The tumor migrating tropism of MSCs has been acknowledged in recent years [26,27,30]. However, hardly any investigation has been done concerning the kinetic distribution of the systematic administration of MSCs in the body, and no consensus exists on how long before the MSCs are first found in the tumor site. Mariam [28] detected MSCs in lung metastases 3 days after intravenous injection, whereas Michael [27] detected MSCs in lung metastases after 20 days. In our study, we investigated the homing ability and distribution pattern of MSCs in a certain course ranging from 24 hours to 72 hours after injection. A considerable number of Adv-Stat3(-)-loaded MSCs were found in the lungs, liver, spleen, and tumor periphery at 24 hours. With time, the number of MSCs in the lungs, liver, and spleen decreased to an extremely low level by 72 hours, whereas the number of MSCs in the tumor increased over this same time period. The localization of MSCs in the lungs, liver, and spleen was possibly determined to be a result of either the abundance of blood flow contributing to passive arrest or the phagocytosis of MSC's by macrophages in these organs. After several cycles of blood circulation, most of the MSCs redistributed to the tumor parenchyma.

In the in vitro experiment, we found Adv- stat3(-) could exponentially amplify in MSCs. After 72 hours' amplification, the Adv- stat3(-) began to make a decreased stat3 expression in the MSCs (data not shown) and eventually released from the carrier at day 4. However, in vivo experiment demonstrated it took no more than 72 hours that most of Adv-Stat3(-)-loaded MSCs have already arrived at the tumor sites. It suggests MSCs could carry a sufficient amount of Adv-Stat3(-) to the tumor sites before they were lysed by the virus.

The possible direct effects that MSCs may have on the host should not be neglected. Some studies have presented intrinsic anti-neoplastic properties in Kaposi's sarcoma [40] and subcutaneous breast tumors [41]. Various explanations have been proposed for these effects, including Akt inhibition [40] and the Wnt pathway [41]. Conversely, Karnoub reported the MSCs promote tumor development in a subcutaneous breast tumor model [42]. Additionally, no positive or negative effect of MSCs was revealed by Sasportas in a glioma model [30]. In our orthotopic breast tumor model, we did not see any increase or decrease in tumor growth following direct MSC injection. The disparate effects of MSCs on tumor growth among these investigations may be ascribed to the different histological tumor types and route of MSC administration. Even if MSCs had a positive effect on tumor progression, the adenovirus released from carrier cells can be anticipated to locally infect and destroy more MSCs, which eliminates the tumor progression influenced by MSCs. Therefore, the beneficial outcomes of CRAd-loaded MSCs in tumor treatment are expected regardless of the positive or negative effects of MSCs on tumor progression.

In our study, an athymic BALB/c mouse model lacking T cells, but having normal B cells, macrophages, and NK cells, was used. In addition to inhibiting tumor growth through tumor oncolysis and suppressing the Stat3 pathway, MSC/Adv-Stat3(-) treatment notably increase the recruitment of CD11b-positive cells in the tumor sites, which include granulocytes and macrophages. Basing on the findings reported by Burdelya [43] and Wang [44], we hypothesize knockdown of stat3 in tumor cells could cause a rise in multiple inflammatory mediators and make the tumor cells more immunogenic that result in recruitment of more CD11b-positive cells from peripheral circulation to the tumor site than the control groups. Some studies have reported infiltrating macrophage could act as an anticancer role [45,46]. In contrast, there were more and more cases in which the presence of infiltrating macrophage was correlated with a poor prognosis [47], which should not be neglected. The above inconsistency could be possibly ascribed to the complexity of the tumor/tumor-associated macrophage (TAM) relationship and the detailed mechanism needs to be clarified in the further investigations.

## Conclusions

This study presents the kinetic process underlying MSC trafficking to tumors and the dynamic course of the amplification and release of E1A-mutant CRAd from MSCs. MSCs not only act as a cell carrier, but also allow the replication of CRAd, significantly enhancing the oncolytic effect and resulting in an augmented tumor inhibition efficiency.

## List of abbreviations

CRAd: conditionally replicative adenovirus; MSC: mesenchymal stem cells; TCID_50,_: 50% tissue culture infective dose; HUVEC: human umbilical vein endothelial cell; RGD: argininine-glycine-aspartic acid; CAR: coxsackie and adenovirus receptor; MOI: multiplicity of infection; PBS: phosphate-buffered saline.

## Competing interests

The authors declare that they have no competing interests.

## Authors' contributions

XX and TJ planned and carried out the cell culture experiments. YF, SL, ZH and JW carried out the TCID_50_, RT-PCR and crystal violet staining. LY, QM and YW carried out in situ hybridization and immunofluorescence staining. XL, PC, KL and LC participated in flow cytometry and western blot. HX, WH, RL, PW and MW carried out and supervised the animal experiments. QG, GX and DW participated in the data interpretation and provided expertise in molecular biological techniques. SW was responsible for writing and revising the manuscript. DM and JZ participated in the design and coordination of the study. All authors have read and approved the final manuscript.

## Supplementary Material

Additional file 1 **Figure 1**. Representative Western blot demonstrating the changes in Stat3 caused by MSC/Adv-Stat3(-), Adv-Stat3(-), and MSC in HUVEC cells. Medium was used as a negative control. Click here for file

Additional file 2 **Figure 2**. MSC preferentially homes to pre-established breast orthotopic tumors. (A) Schematic graph showing the design of the experiment. (B) Representative images showing the distribution of MSCs in the tumor. 1 × 10^6 ^SPIO-labeled MSCs were intravenously injected into tumor-bearing mice. The mice were sacrificed and frozen sections prepared at 24 hours (upper panels) and 72 hours (lower panels). Prussian blue staining was performed to detect the presence of MSCs in the tumor (left columns, black arrows), and the sections were counterstained with nuclear fast red. Sequential sections stained with H&E are shown in the right columns. (C) Representative images showing the distribution of MSCs in normal tissues. 72 hours after intravenous injection of 1 × 10^6 ^SPIO-labeled MSCs, the mice in panel b were sacrificed and their organs prepared for frozen sections followed by Prussian blue and nuclear fast red staining. B, scale bar = 10 μm; C, scale bar = 20 μm. Click here for file
