# Supplementary figures and images for: Mesenchymal stem cells as carriers and amplifiers in CRAd delivery to tumors
Source: Mol Cancer. 2011 Nov 3;10:134. doi: 10.1186/1476-4598-10-134 (PMC3215929; doi:10.1186/1476-4598-10-134)

Medium

MSC

Adv-stat3(-)

MSC/Adv-stat3(-)

Stat3

$\beta$ -actin

HUVEC

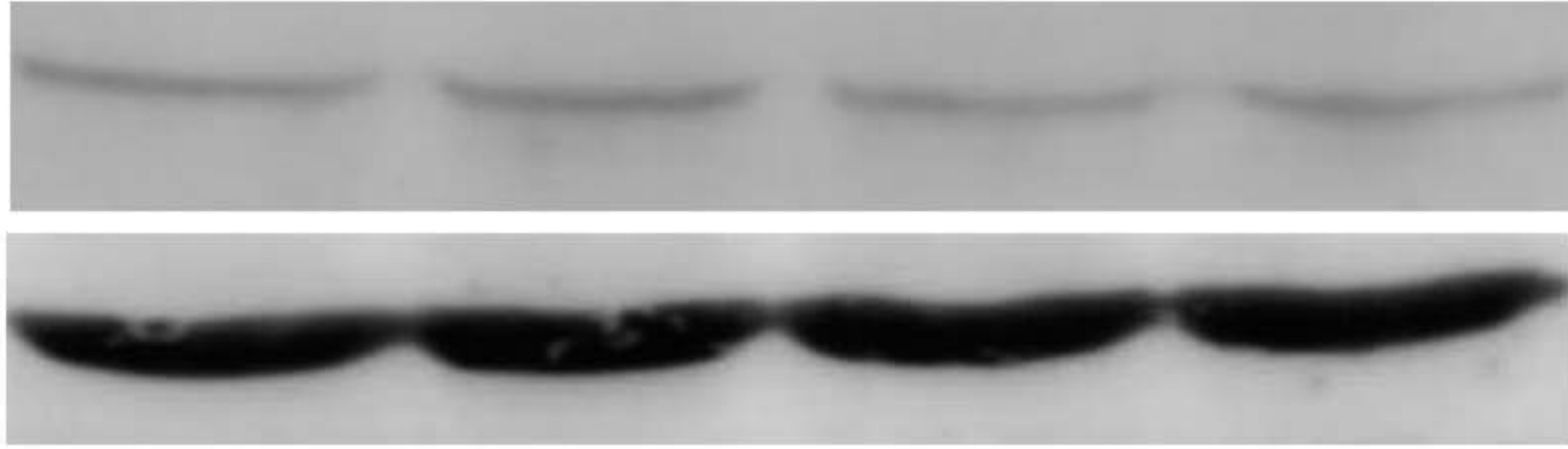

Supplement: Additional file 1 — Figure 1. Representative Western blot demonstrating the changes in Stat3 caused by MSC/Adv-Stat3(-), Adv-Stat3(-), and MSC in HUVEC cells. Medium was used as a negative control. [file 1476-4598-10-134-S1.PDF]

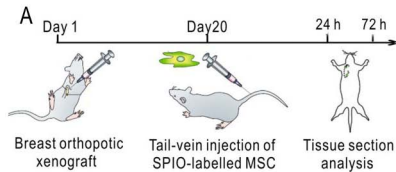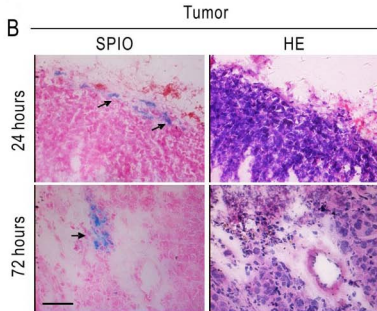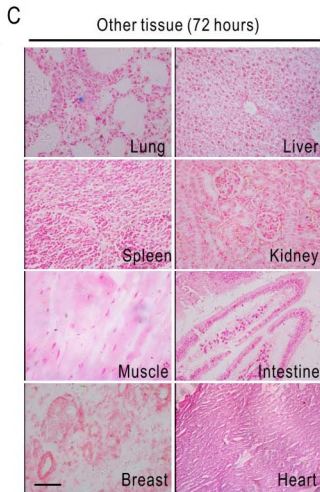

Supplement: Additional file 2 — Figure 2. MSC preferentially homes to pre-established breast orthotopic tumors. (A) Schematic graph showing the design of the experiment. (B) Representative images showing the distribution of MSCs in the tumor. 1 × 10^6^ SPIO-labeled MSCs were intravenously injected into tumor-bearing mice. The mice were sacrificed and frozen sections prepared at 24 hours (upper panels) and 72 hours (lower panels). Prussian blue staining was performed to detect the presence of MSCs in the tumor (left columns, black arrows), and the sections were counterstained with nuclear fast red. Sequential sections stained with H&E are shown in the right columns. (C) Representative images showing the distribution of MSCs in normal tissues. 72 hours after intravenous injection of 1 × 10^6^ SPIO-labeled MSCs, the mice in panel b were sacrificed and their organs prepared for frozen sections followed by Prussian blue and nuclear fast red staining. B, scale bar = 10 μm; C, scale bar = 20 μm. [file 1476-4598-10-134-S2.PDF]
